# Elaborating the molecular characteristics of corals’ different tolerance to environmental stress in Sanya Luhuitou based on multi-omics analysis

**DOI:** 10.3389/fmicb.2025.1664176

**Published:** 2026-01-06

**Authors:** Xiaoyu Tang, Xiangrui Guo, Hanzhang Wang, Qingsong Yang, Ying Zhang, Juan Ling, Hao Sun, Junde Dong, Yanying Zhang

**Affiliations:** 1 CAS Key Laboratory of Tropical Marine Bio-Resources and Ecology, Guangdong Provincial Key Laboratory of Applied Marine Biology, South China Sea Institute of Oceanology, Chinese Academy of Sciences, Guangzhou, China; 2 Ocean School, Yantai University, Yantai, China; 3 Guangdong Provincial Observation and Research Station for Coastal Upwelling Ecosystem, South China Sea Institute of Oceanology, Chinese Academy of Sciences, Shantou, China; 4 Sanya National Marine Ecosystem Research Station, Tropical Marine Biological Research Station in Hainan, Chinese Academy of Sciences, Sanya, China

**Keywords:** coral, bacteria, transcriptome, proteome, metabolome, environmental stress tolerance

## Abstract

**Introduction:**

The resistance to environmental perturbations varies significantly among coral species. Corals are holobionts that are symbiotic with dinoflagellates and microbiomes, which makes their physiological responses to environmental stress complex. In order to restore coral reefs, it is essential to discover the molecular characteristics associated with coral environmental stress tolerance and to understand the molecular mechanisms that contribute to physiological adaptation.

**Methods:**

Using high throughput 16S rRNA gene sequencing, combined with transcriptome, proteome, and metabolome analyses, we analyzed the differences in coral associated bacterial communities between the branching coral (*Pocillopora damicornis*) and massive corals (*Porites lutea* and *Galaxea fascicularis*), as well as the profiling of environmental stress resistance related genes, proteins and metabolites in these coral species.

**Results:**

The results showed that beneficial bacteria were more abundant in massive corals than in branching corals, while pathogenic bacteria were more abundant in branching corals. Genes and proteins that can counteract environmental stress were found more abundant in branching corals as compared to massive corals. Branching corals contained higher levels of metabolites associated with environmental stress, such as LysoPC (15:0). Massive corals possess simultaneously higher basal expression genes (or proteins) involved in amino acid metabolism, which may contribute to their higher tolerance.

**Discussion:**

Based on molecular characteristics, branching corals’ resistance to environmental stress was weaker than that of massive corals, which provided a valuable reference for coral reef protection in the future.

## 1 Introduction

Coral reefs are one of the most important ecosystems on earth and are usually deemed as tropical rainforests in the ocean because of their high primary productivity and biodiversity (Hughes et al., 2002; Roberts et al., 2002). Coral reefs can provide many ecosystem services for human beings, including food provision, fisheries and tourism, as well as coastal protection against natural hazards (França et al., 2020). Despite occupying only 0.2% of the ocean’s surface, coral reefs are home to at least 25% of all known marine organisms (Harvey et al., 2018; Vanwonterghem and Webster, 2020). However, marine heatwaves lead to the water temperature abnormally warm relative to seasonal variations, which damaged marine ecosystems and global biodiversity heavily (Hobday et al., 2016; Smale et al., 2019; Byrne et al., 2025). Global marine heatwaves severely affected coral reef ecosystems worldwide, causing coral bleaching and mortality (Morrison et al., 2020; Sun et al., 2020; Mellin et al., 2024). Also, human perturbations such as overharvesting, pollution discharge, and coastal development are driving coral reefs into rapid decline, with the rate of decline for many coral reefs accelerating in recent decades (Van Oppen et al., 2017; Hoegh-Guldberg et al., 2018; Staples and Pandolfi, 2024). As a result, corals’ ability to adapt to environmental perturbations is critical to the survival of coral reefs under the pressure of global climate change.

Both experimental studies and field investigations have found that the susceptibility of corals to environmental stress appears to vary distinctly among species (Marshall and Baird, 2000; Wooldridge, 2014; García et al., 2024; Long et al., 2024; Lucey et al., 2024). As an example, in the Sanya fringing reef, South China Sea (SCS), the branching coral *Pocillopora* was more susceptible to heat bleaching than the massive coral *Porites* (Li et al., 2008). *Galaxea* was found to be among the least susceptible taxa on the Great Barrier Reef (Marshall and Baird, 2000). Physiological responses of corals to environmental stress are complex since corals are holobionts that are symbiotic with dinoflagellates and microbiomes (Reshef et al., 2006; Hsieh et al., 2024; Marangon et al., 2025). The tolerance of corals to environmental stress is influenced by a number of factors, such as environmental memory (Hackerott et al., 2021), coral colony morphology and size (Van Woesik et al., 2012), coral growth form (Chou et al., 2016), and genetic diversity (Richards et al., 2023).

The coral holobiont copes with environmental stresses (e.g., thermal stress, acidification, eutrophication) via a multi-level regulatory network involving gene expression, protein activation, and metabolic reprogramming. During thermal stress, heat shock proteins (HSPs) in coral hosts are overexpressed (Li et al., 2021), and genes related to coral immune responses are upregulated (Helgoe et al., 2024). In Symbiodiniaceae, the expression of genes associated with photosynthesis, metabolism, antioxidant activity, and immune responses changes (Gierz et al., 2017), triggering a heat stress cascade in the coral holobiont. This cascade leads to the excessive production of reactive oxygen species (ROS), which are released into coral host cells. The excessive production and toxic accumulation of ROS cause damage to both coral hosts and symbiotic algae (Doering et al., 2023). Meanwhile, under environmental stress, Symbiodiniaceae retain more organic carbon for their own utilization, leading corals into a state of “carbon limitation” (Cui et al., 2023; Rädecker et al., 2023), and the symbiotic relationship between corals and Symbiodiniaceae is disrupted (Suggett and Smith, 2020; Doering et al., 2023). Additionally, the dynamics of coral bacterial communities are related to their tolerance of environment (Ziegler et al., 2019; Yu et al., 2020; Chen et al., 2024). However, the potential molecular characteristics have not been fully elucidated. Single-omics approaches such as transcriptomic, proteomic, or metabolomic, are commonly used to characterize the gene expression profiles, protein expression patterns, and metabolite signatures of corals under environmental stress (Barshis et al., 2010; Bay and Palumbi, 2017; Greene et al., 2020; Lima et al., 2024). In contrast, multi-omics integrative analyses for dissecting interspecific differences in environmental tolerance among coral species are key to deciphering coral resilience. Given the mass coral bleaching and mortality caused by environmental stress, revealing the molecular characteristics related to coral environmental tolerance and understanding the molecular mechanisms behind physiological adaptation are essential for the establishment of coral reef models in the upcoming climate change scenarios (Palumbi et al., 2014; Cropp and Norbury, 2020; Mayfield and Lin, 2023).

Coral thermal bleaching was observed in Luhuitou, Sanya in 2010, 2015, and 2017 (Huang et al., 2021). After thermal bleaching events, however, the intrinsic molecular characteristics of the differences in bacterial communities, gene expression, proteins, and metabolites among the dominant coral species include *Pocillopora damicornis* (PD), *Porites lutea* (PL), and *Galaxea fascicularis* (GF) under normal growth conditions (without artificial stress treatment) is unknown. We hypothesize that different coral species exhibit distinct molecular characteristics associated with environmental stress. This study evaluates the resistance and resilience of the selected dominant coral species to environmental stress, thereby providing a reference for the future environmental tolerance of coral reefs in Sanya Bay.

## 2 Materials and methods

### 2.1 Sample collection

In the present study, we collected coral *Pocillopora damicornis* (PD), *Porites lutea* (PL) and *Galaxea fascicularis* (GF) from Luhuitou fringing reef in Sanya, China (18°12′19′′N, 109°28′27′′E) on June 7, 2021. Each coral species included three biological replicates from different individuals (approximately 10 meters apart from each other). The depth of coral collected was 1.5–2 m. The seawater temperature was 27–28 °C, and the salinity was approximately 34‰. Coral samples collected were immediately snap frozen in liquid nitrogen and transformed to −80 °C until DNA extraction.

### 2.2 DNA extraction, Illumina MiSeq sequencing and analysis

Total genomic DNA was isolated using the E. Z. N. A.® soil DNA Kit (Omega, United States). PCR amplification of 16S rRNA genes (V3–V4) was performed following the methods detailed in our previous study (Tang et al., 2024). We constructed a sequencing library using equal molar concentrations of purified PCR products. A paired-end sequence was performed on an Illumina HiSeq 2500 platform by Gene-Denovo (Guangzhou). Raw sequencing reads were then deposited into the NCBI Sequence Read Archive (SRA).

Following demultiplexing, the sequences were combined and then subjected to quality filtration through fastp (v0.19.6) (Chen et al., 2018), with longer sequences (>275 bp) and sequences containing ambiguous base pairs were removed. In order to further reduce noise, high-quality sequences were subjected to noise reduction using the DADA2 plugin within Qiime2 (version 2020.2) (Bolyen et al., 2019). The amplicon sequence variants (ASVs) were then generated. The number of sequences from each sample was rarefied to 4,000. The taxonomic classification of ASVs was carried out using the Naïve Bayes consensus taxonomy classifier, along with the SILVA 16S rRNA database (v138). Principal co-ordinates analysis (PCoA) was performed based on the abundance of ASV of each sample. Analysis of similarities (ANOSIM), Permutational Multivariate Analysis of Variance (Adonis) and Multiple Response Permutation Procedure (MRPP) were used to test the differences in microbiomes of three coral species.

### 2.3 RNA extraction, sequencing and transcriptomic data processing

Following the previously described methods (Zhang et al., 2024), we extracted total RNA using the Trizol reagent (Invitrogen, Carlsbad, CA, United States). We evaluated the quality of the isolated RNA using an Agilent 2100 Bioanalyzer (Agilent Technologies, Palo Alto, CA, United States). Hieff NGS Ultima Dual-mode mRNA library preparation kit (Shanghai Yeasen Biotechnology Co., Ltd., Shanghai, China) was used for library preparation. The prepared libraries were then sequenced on the Illumina HiSeq™ 4000 system, provided by Gene Denovo Biotechnology Co. (Guangzhou, China).

SeqPrep^1^ and Sickle^2^ were used with default parameters to adapter trim and quality filter raw sequencing reads. Bowtie2 (version 2.2.8) (Langmead and Salzberg, 2012) was employed to map reads against ribosomal RNA (rRNA) databases, with the resulting rRNA-mapped reads subsequently removed. An alignment to the reference genome (Supplementary Table S1) was conducted using HISAT2 in strand-specific mode using reference-guided alignment (Kim et al., 2015). A reference-based transcript assembly approach was then applied using StringTie (v2.2.1) and reads per kilobase per million mapped reads (RPKM) was used to estimate gene expression (Shumate et al., 2022). A differential expression analysis was conducted using DESeq2 (Love et al., 2014). We identified significant differentially expressed genes (DEGs) with |log2FC| > 1 and false discovery rate (FDR) < 0.05.

Transcriptome analysis of Symbiodiniaceae was performed using *de novo* assembly following our previous method (Zhang et al., 2024). The assembled unigenes were mapped to the above-indexed Symbiodiniaceae reference genome via HISAT2 (Kim et al., 2015); finally, the mapped unigenes were calculated and normalized to RPKM (Li and Dewey, 2011).

Kyoto Encyclopedia of Genes and Genomes (KEGG) functional enrichment analysis was used to identify DEGs significantly enriched in metabolic pathways (Bonferroni-corrected *p*-values ≤ 0.05 compared with the whole-transcriptome background). Goatools^3^ was used to perform the KEGG pathway analysis.

### 2.4 Protein extraction and analysis

Using a buffer containing 7 M urea, 2 M thiourea, and 1% SDS, we extracted total protein from coral samples. Protein concentrations were determined using a BCA protein assay. Fifty microgram of protein was transferred into a new Eppendorf tube and the final volume was adjusted to 50 μL with 1 M DTT 1 μL and incubated at 55 °C for 1 h. The sample was incubated for 1 h at room temperature in 5 μL of 1 M IAA. Five volumes of −20 °C pre-chilled acetone was added to precipitate the proteins overnight. After washing twice with a pre-chilled 90% acetone aqueous solution, precipitates were resuspended in ammonium bicarbonate (50 mM). The proteins were digested overnight at 37 °C with sequence grade modified trypsin (Promega, Madison, WI) at a ratio of 1:50 (enzyme:protein, weight:weight).

We fractionated peptide mixtures using reversed-phase separation at high-pH. Upon completion of fractionation, solvent A, containing 0.1% formic acid in water, was used to re-dissolved the peptides. We then used an Orbitrap Fusion Lumos coupled with an EASY-nLC 1200 system (Thermo Fisher Scientific, MA, United States) for online nanospray LC–MS/MS. The peptide sample was loaded onto an analytical column (Acclaim PepMap C18, 75 mm × 25 cm). The column was eluted with a 120-min gradient, where the proportion of solvent B increased from 5 to 35%. SolventB contained 0.1% formic acid in acetonitrile. Flow rate and temperature of the analytical column was 200 nL/min and electrodespray ionization was performed at a voltage of 2 kV in relation to the mass spectrometer’s inlet. A data-independent acquisition (DIA) mode was used to acquire mass spectrometry data, allowing the analysis of peptide ions to be comprehensive and unbiased.

Spectronaut X (Biognosys AG, Switzerland) was used to process DIA data with default settings against coral and Symbiodiniaceae protein database obtained from RNA-seq results. Quantification was conducted on all precursors that passed filters. Major group quantities were calculated by averaging the top three filtered peptides that passed a 1% *Q* value threshold. To identify differentially expressed proteins (DEPs), Student’s *t*-test was used. We used two-fold change thresholds and *Q*-value < 0.05 to identify the DEPs. The KEGG database was used to annotate all identified proteins, and the DEPs were then used to analyze KEGG pathways.

### 2.5 Metabolite extraction and analysis

For metabolite extraction, 100 μL of sample was mixed with 300 μL of methanol and 20 μL of internal standard, vortexed for approximately 30 s, and subjected to ultrasonic extraction in an ice-water bath for 5 min. The mixture was then stored at −20 °C for 2 h, followed by centrifugation at 13,000 rpm and 4 °C for 15 min. A 200 μL aliquot of the supernatant was transferred to a 2 mL sample vial for subsequent LC–MS analysis. Quality control (QC) samples were prepared by pooling equal volumes of extracts from all experimental samples to assess the repeatability of sample processing. During instrument analysis, one QC sample was inserted every 6 test samples to monitor the stability of the entire analytical process.

Metabolite detection was performed using a UHPLC system coupled with a Q Exactive Orbitrap high-resolution mass spectrometer, which acquired both primary and secondary mass spectrometry data. Mobile phases were optimized for ion modes: in positive mode, 0.1% formic acid aqueous solution (A) and acetonitrile (B); in negative mode, 5 mM ammonium acetate aqueous solution (pH adjusted to 9.0 with ammonia water, A) and acetonitrile (B). QC samples were analyzed using the Full MS-ddMS^2^ method with four segmented scan ranges (70–200 m/z, 190–400 m/z, 390–600 m/z, and 590–1,000 m/z), each scanned once to enhance secondary data acquisition for metabolite identification. The raw data obtained after mass spectrometry analysis were processed through baseline filtering, peak recognition, integration, retention time correction, peak alignment, and normalization, resulting in a data matrix containing retention time, mass-to-charge ratio, and peak intensity. Metabolites were qualitatively identified by comparing against public databases like the Human metabolome database (HMDB) and Metlin (Smith et al., 2005; Wishart et al., 2018).

Orthogonal partial least squares discriminate analysis (OPLS-DA) was performed to determine global metabolic changes between comparable groups using the ropls R package from Bioconductor (Kang et al., 2022). Model validity was verified via cumulative determination coefficients (R^2^) and cross-validation parameters (Q^2^). Although the potential limitation of inflating group separation, the significant metabolites were identified using a dual-threshold screening strategy: variable importance in projection (VIP) scores > 1.0 from OPLS-DA models combined with paired t-test *p*-values < 0.05. Metabolites that were different between the two groups were identified. The biochemical pathways of these metabolites were then mapped. KEGG was used for metabolic enrichment and pathway analysis (Chen et al., 2016).

### 2.6 mRNA and protein correlation analyses

An analysis of the association between genes and proteins was conducted quantitatively. The transcriptome and proteome were analyzed separately for genes and proteins exhibiting differential expression. A nine-quadrant map analysis was used to identify DEGs/DEPs that changed consistently in GF and PL (Wang et al., 2022). The analysis was conducted using R (version 3.5.1, R Core Team, 2018). To further analyze if the coral holobionts also differed with respect to functional or molecular categories, we carried out KEGG pathway enrichment analysis using a threshold value (*Q* < 0.05) based on DEGs/DEPs with significantly concordant changes.

## 3 Results

### 3.1 Diversity and community assembly of bacterial community in three coral species

**Figure 1:**
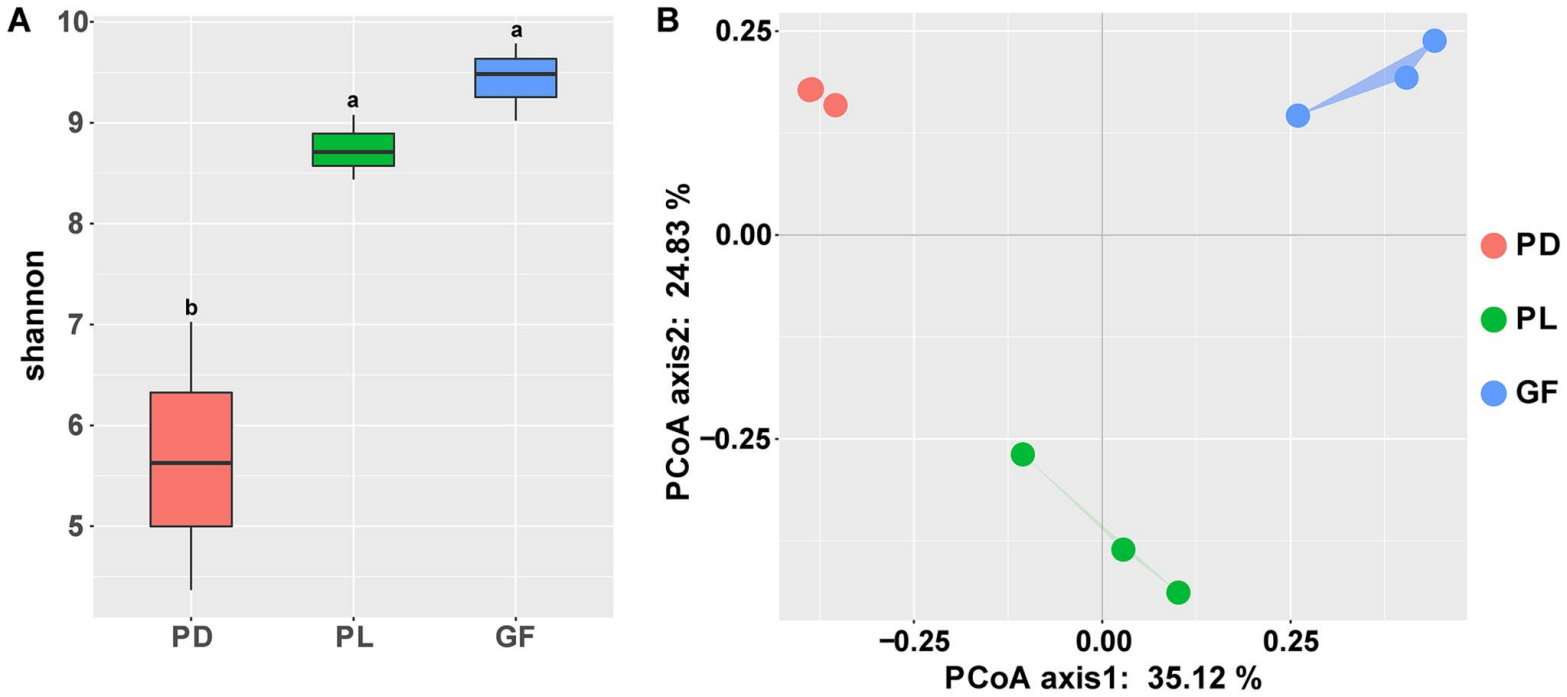
Diversity analysis of bacterial communities associated with *Pocillopora damicornis* (PD), *Porites lutea* (PL), and *Galaxea fascicularis* (GF): **(A)** Alpha diversity and **(B)** principal co-ordinates analysis. Different letters above the box indicate significant dissimilarity determined through one-way analysis of variance (ANOVA).

According to the rarefaction curve, all coral sample showed a sufficient coverage (Supplementary Figure S1). Alpha diversity in bacterial communities associated with three coral species differed, with *Pocillopora damicornis* (PD) having a significantly lower Shannon index than the other two coral species (*p* < 0.05, Figure 1A). However, there was no significant difference between the Shannon indexes of *Porites lutea* (PL) and *Galaxae fascicularis* (GF) (*p* > 0.05). Other alpha diversity indices of the coral-associated bacterial community a similar pattern to the Shannon index (Supplementary Table S2). The results of principal co-ordinates analysis (PCoA) of the bacterial community indicated that the coral-associated bacterial community was significantly different among three coral species (Figure 1B). These differences were substantiated by three difference tests including ANOSIM, Adonis and MRPP (*p* < 0.01), which all corroborated the results with the PCoA (Supplementary Table S3).

**Figure 2:**
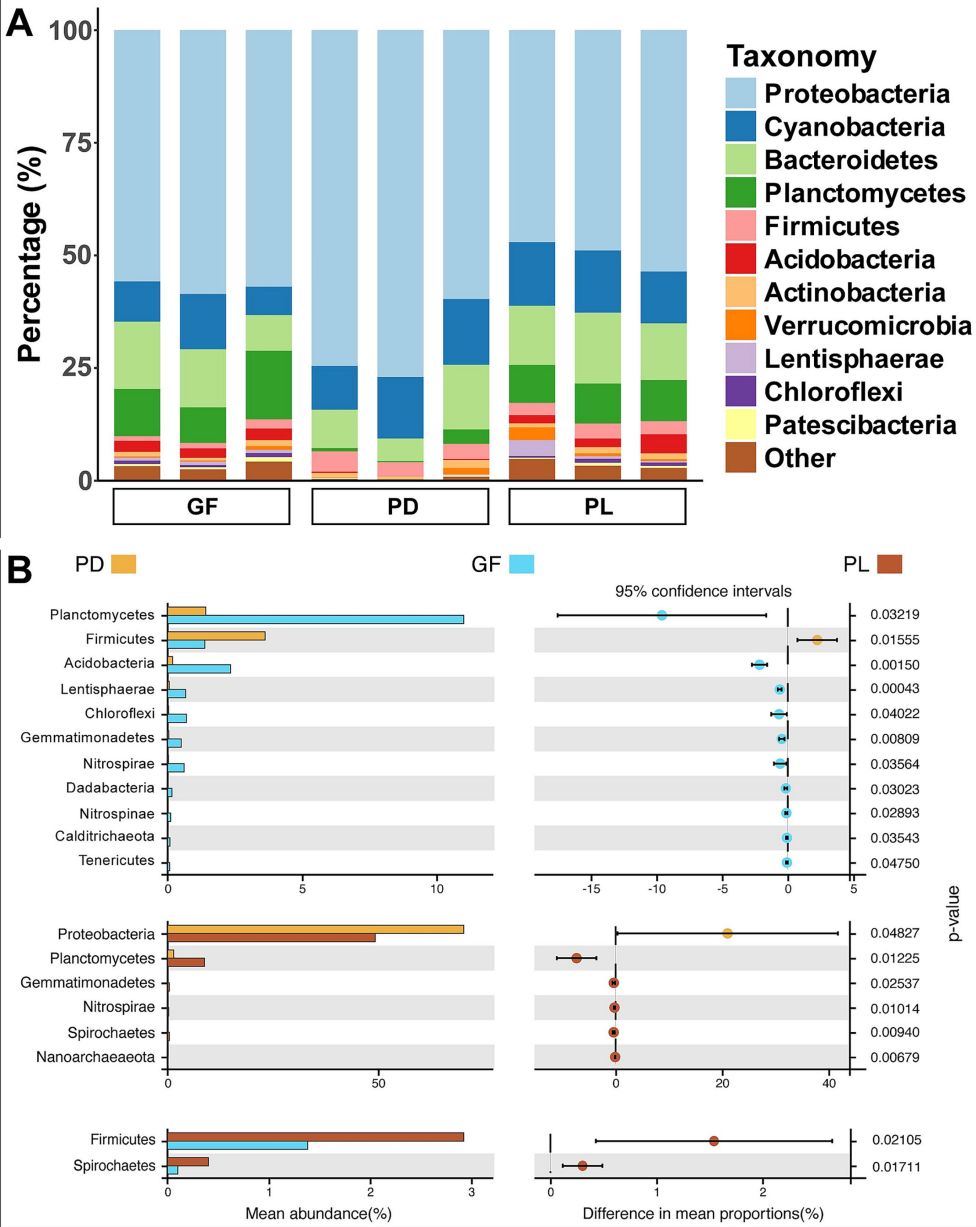
The taxonomic dynamics of bacterial communities associated with PD, PL, and GF. **(A)** Bacterial community composition at phylum level in PD, PL, and GF. **(B)** Differences of bacterial community at phylum level between PD, PL, and GF. The phyla with lower abundance than the top 12 in each coral species were classified as “Other”.

Taxonomic classification demonstrated that Proteobacteria was the dominant group among the bacterial communities associated with all coral samples, accounting for 70.6% in PD, 49.9% in PL and 57.0% in GF, respectively (Figure 2A). We found that six phyla, including Proteobacteria, Planctomycetes, Gemmatimonadetes, Nitrospirae, Spirochaetes, and Nanoarchaeaeota, had significantly different abundances between PD and PL, and 11 phyla were significantly different between PD and GF (*p* < 0.05, Figure 2B). Also, the abundances of Planctomycetes, Gemmatimonadetes and Nitrospirae in PD were significantly lower than in PL and GF. Between PL and GF, only two phyla (Firmicutes and Spirochaetes) showed significantly differences (*p* < 0.05). At the genus level, the abundances of *Acinetobacter*, *Endozoicomonas*, *Ralstonia*, and *Sphingomonas* in PD were statistically significantly higher than those of PL and GF (*p* < 0.05, Supplementary Figure S2). By contrast, the abundance of *Ruegeria*, *Chlorobium* and *Pelagibius* in PD was lower than that of PL and GF (*p* < 0.05). As compared to PD, the bacteria communities of PL and GF had a more similar composition with the abundance of the top 11 genus in PL and GF being higher or lower than that of PD except the *Fulvivirga*.

### 3.2 Differentially expressed genes among coral host and Symbiodiniaceae

*De novo* assembled transcriptomes from three coral species and associated Symbiodiniaceae contain 112,655 and 36,938 putative genes, respectively. Initial analysis showed that PDGF (PD versus GF) was an outlier, with 64,740 DEGs detected in the coral host transcriptome compared to 82,227 and 83,555 in PDPL (PD versus PL) and PLGF (PL versus GF), respectively (Supplementary Table S4). Compared with PL, a total of 37,185 genes in PD exhibited higher basal expression, while 45,042 genes showed lower basal expression. Compared with GF, PD also exhibited 32,838 genes with significantly higher basal expression and 31,902 genes with significantly lower basal expression. Like the coral host, differential gene expression indicated that PDGF was an outlier, with 1,535 DEGs in the Symbiodiniaceae transcriptome compared to 36,503 and 36,353 in PDPL and PLGF, respectively. Detailly, the majority of DEGs (975 or 64%) showed significantly lower basal expression in PD relative to GF. Compared with PL, 18,543 genes in PD showed significantly higher basal expression, while 17,960 genes exhibited significantly lower basal expression. When comparing PL with GF, 17,820 genes in PL had significantly higher basal expression, whereas 18,533 genes had significantly lower basal expression.

**Figure 3:**
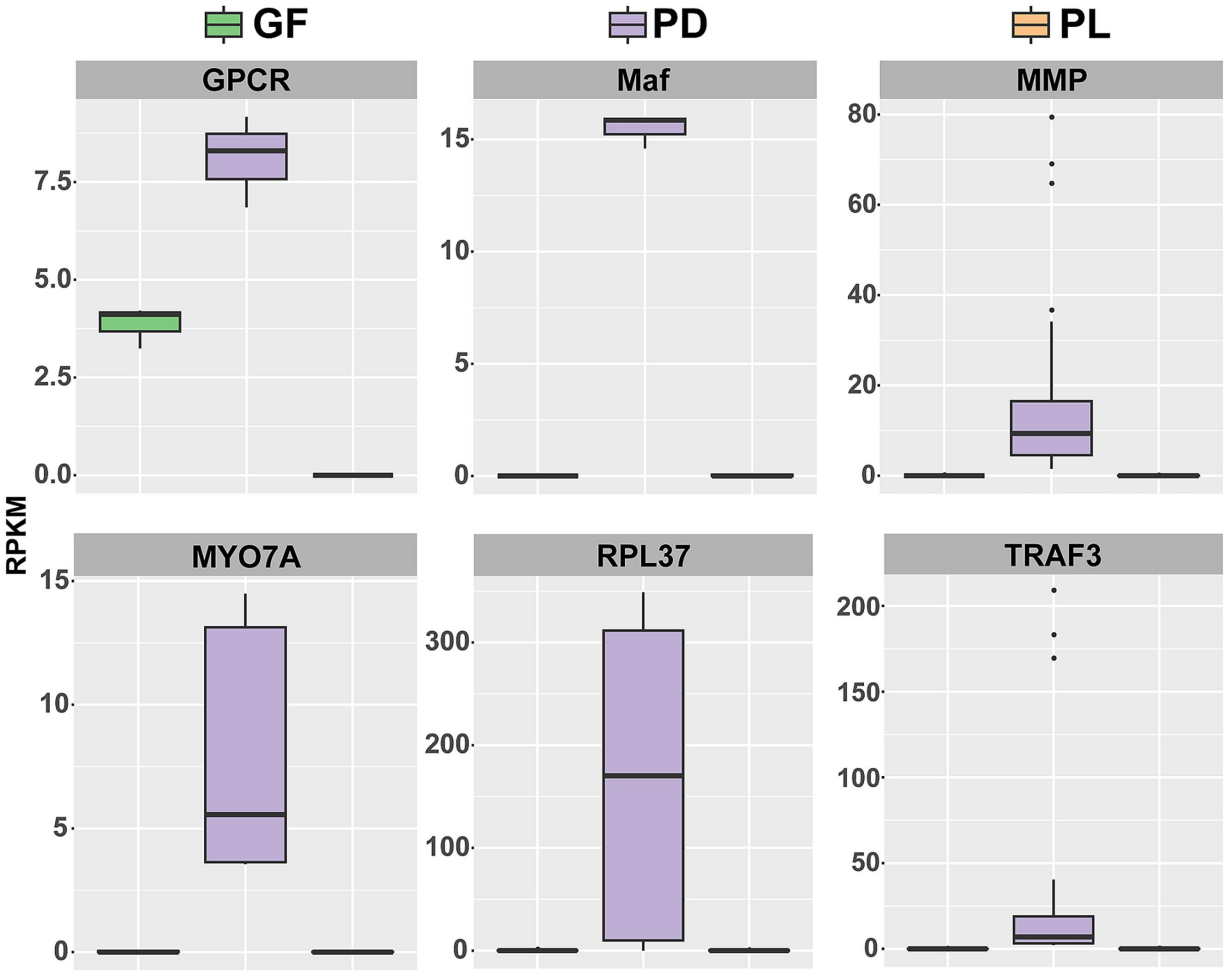
The abundance of same expressed environmental-stress related DEGs in PD, PL, and GF.

Twenty-four environmental-stress related DEGs were found to be shared between the PDGF and PDPL groups (Supplementary Table S5). Eight genes shared the same expression pattern (i.e., all exhibit higher basal expression): Maf, matrix metalloproteinase (MMP), G protein-coupled receptor (GPCR), myosin VIIA (MYO7A), ribosomal protein L37 (RPL37), tumor necrosis factor receptor associated factor 3 (TRAF3), peroxidasin (PXDN), and elongation factor 1α (EF1α) (Figure 3; Supplementary Table S6). Furthermore, we found a similar expression pattern for thermal stress-related genes between PL and GF.

### 3.3 Differences in the proteomes among three coral host and Symbiodiniaceae

**Figure 4:**
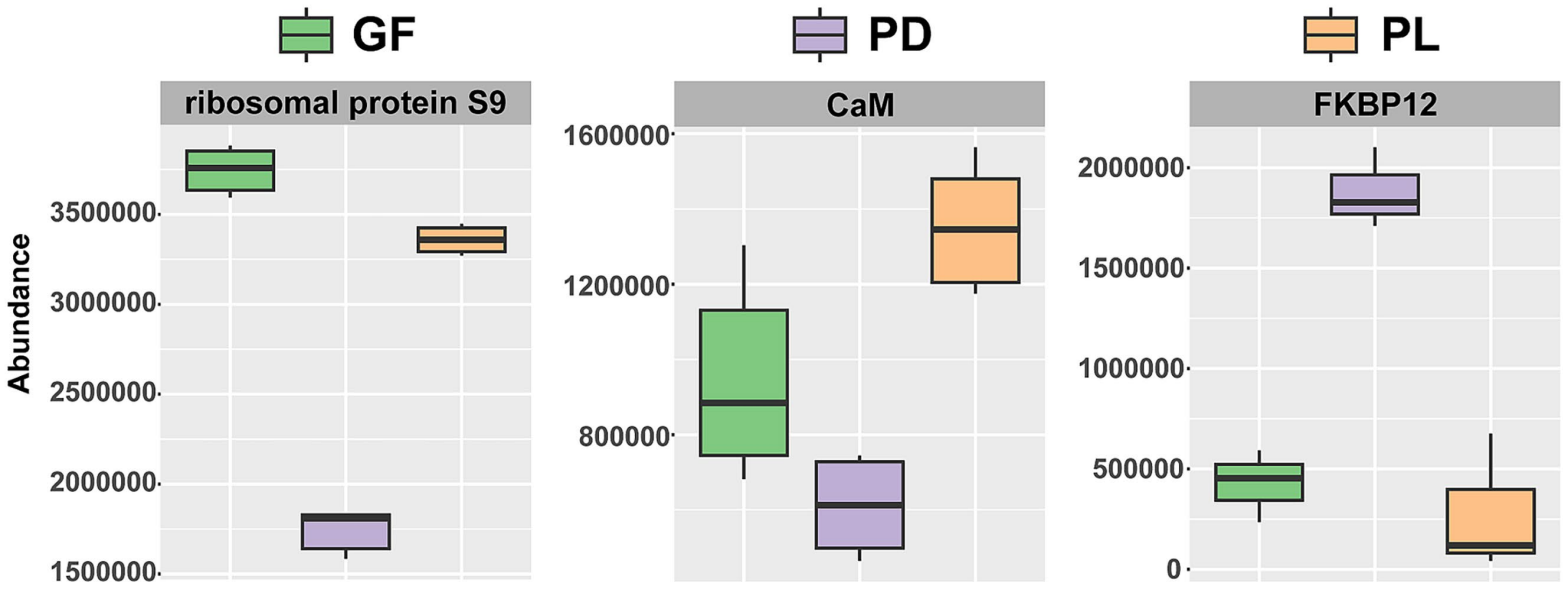
The abundances of environmental-stress related DEPs with the same expression profile in PD, PL, and GF.

A proteomics analysis revealed that most of the proteins expressed were in the range of 10 to 25 kDa, and 16,485 and 626 proteins were identified for the coral host and Symbiodiniaceae, respectively. Differentially expressed proteins (DEPs) were identified based on the selection criterion of a ≥2-fold change at *Q* value < 0.05. Then, 492, 574, and 449 proteins were identified as DEPs in PDPL, PDGF, and PLGF groups for the coral host, respectively (Supplementary Tables S7, S8). The vast majority of DEPs in the PDPL group (331 or 67%) and PDGF group (326 or 57%) exhibited lower basal expression in PD. Particularly, fewer DEPs (207 or 46%) were showed lower basal expression in PL compared to GF. In the proteome of Symbiodiniaceae, a total of 30, 31, and 17 proteins were found to be DEPs in the PDPL, PDGF, and PLGF groups, respectively. Compared with GF and PL, a greater number of DEPs exhibited higher basal expression in PD (16 and 18 in PDPL and PDGF, respectively). In the PLGF group, 11 proteins in PL had higher basal expression relative to GF. The PDGF and PDPL groups shared five thermal-stress related DEPs. Among those, three DEPs had the same expression profile: ribosomal protein S9, calmodulin (CaM), and FK506-binding protein 12 (FKBP12) (Figure 4; Supplementary Table S9).

### 3.4 Metabolic profiling analysis of three coral holobionts

**Figure 5:**
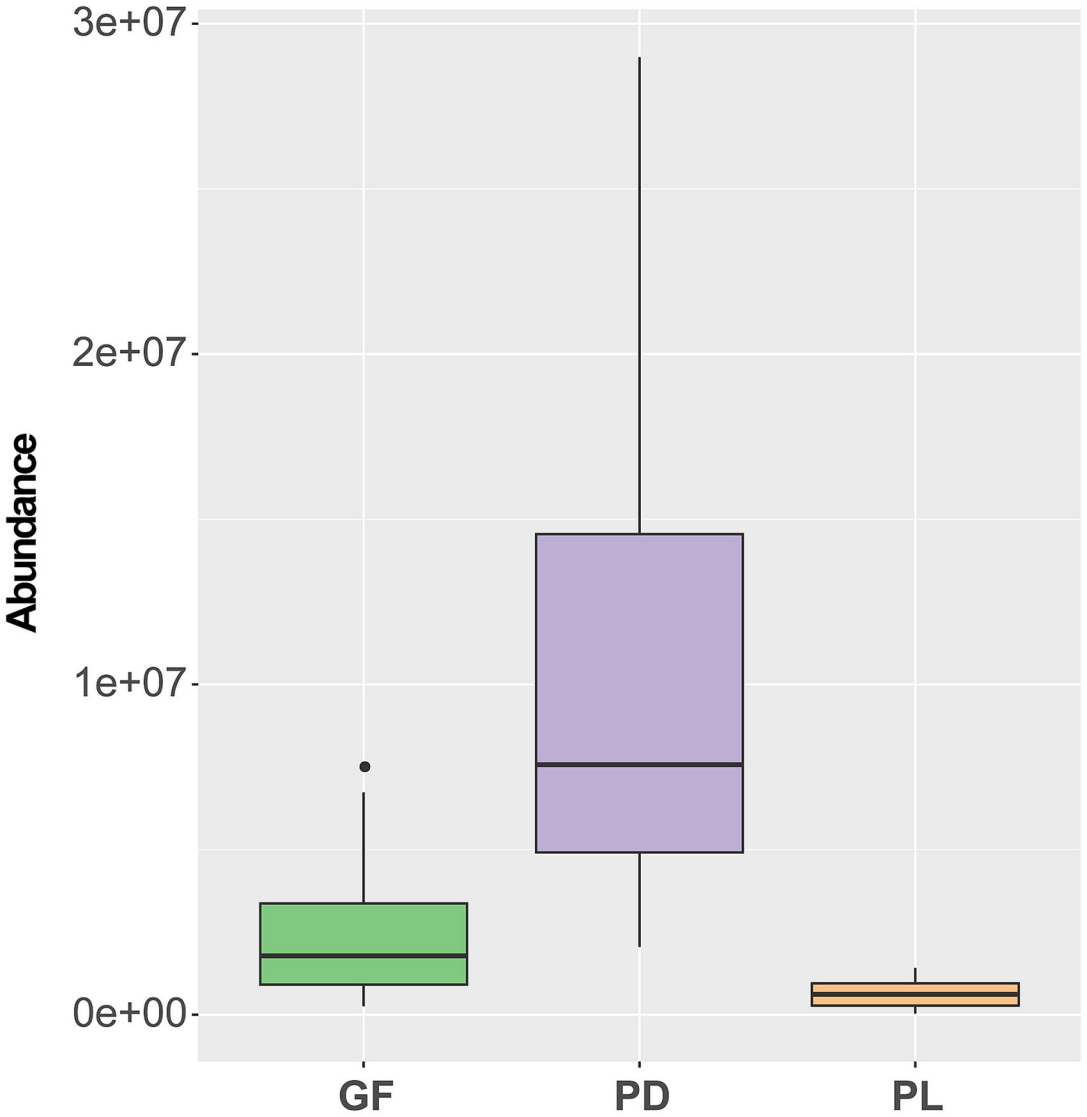
The abundances of LysoPC among three coral species.

Based on metabolic profiling analysis of three coral holobionts, we found that 778, 764, and 607 metabolites were significantly differentially expressed in the PDPL, PDGF and PLGF groups, respectively (Supplementary Tables S10, S11). More than half of the differentially expressed metabolites exhibited higher basal expression in PD compared with PL and GF (457 and 401, respectively). However, more differentially expressed metabolites exhibited higher basal expression in PL compared with GF (385 or 63%). Several metabolites including cholesterol sulfate and testololactone were enriched in PD (Supplementary Table S12). However, deoxycytidine and L-glutamine were found to be significantly higher in GF compared with PD, a similar pattern of deoxycytidine was found in PL. Although not significantly, LysoPC was more abundant in PD than in GF and PL (Figure 5).

### 3.5 Correlation between the transcriptome and the proteome

**Figure 6:**
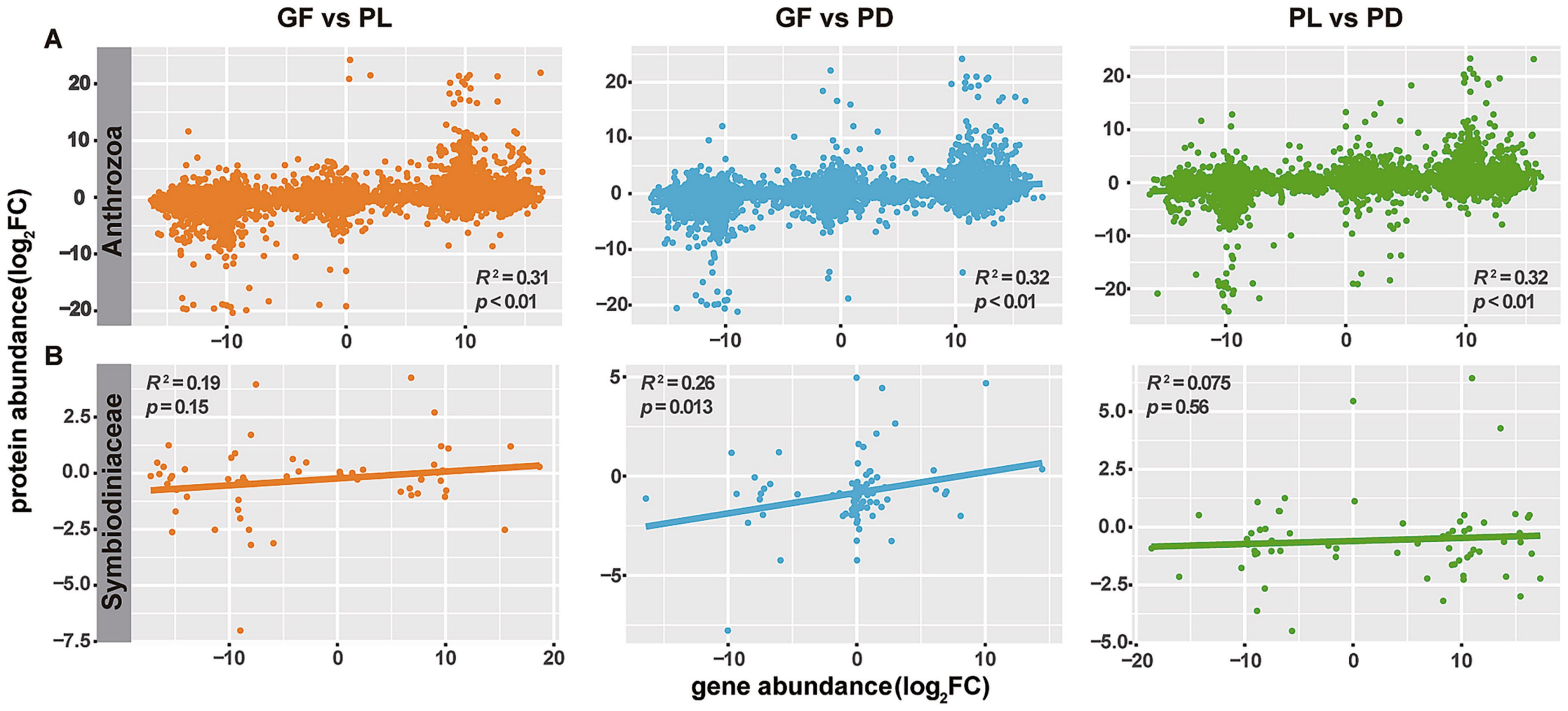
Correlation of protein profile with gene expression for coral host **(A)** and *Symbiodiniaceae* **(B)** samples of PD, PL, and GF, respectively.

We analyzed the correlation between gene and protein expression for coral host and Symbiodiniaceae, the results showed that a similarly weak but highly significant correlation (*p*-value < 0.01) across all coral hosts (Figure 6). A weak and insignificant correlation was found between genes and proteins of Symbiodiniaceae in the PDPL and PLGF groups. Accordingly, coral species do not affect the correlations between genes and proteins directly. Furthermore, we assessed the correlation between DEGs and DEPs for coral hosts and Symbiodiniaceae (Supplementary Figure S3). Interesting findings showed that the previously identified weak correlation was enhanced for coral hosts and Symbiodiniaceae. We hypothesized that the proteins with no significant difference between coral species could drive the weak correlation. It was probable that DEPs would have corresponding genes differentially enriched in the same direction. So, the removal of non-significantly expressed proteins resulted in a higher correlation.

Furthermore, we investigated the association of genes and proteins through nine-quadrant associate analysis and found that more genes (11 or 73%) exhibited lower basal expression in GF for the PLGF group of the coral host (Supplementary Figure S4). Oppositely, only a small number of genes (or proteins) exhibited higher basal expression in PD for the PDGF (11 or 16%) and PDPL (18 or 16%) groups. Few genes (or proteins) had uniformly significant changes in the Symbiodiniaceae samples. Only 1 gene exhibited significantly lower basal expression in PD for the PDGF group, while 1 gene had significantly higher basal expression in PD for the PDPL group.

### 3.6 Function investigation based on KEGG enrichment analysis

**Figure 7:**
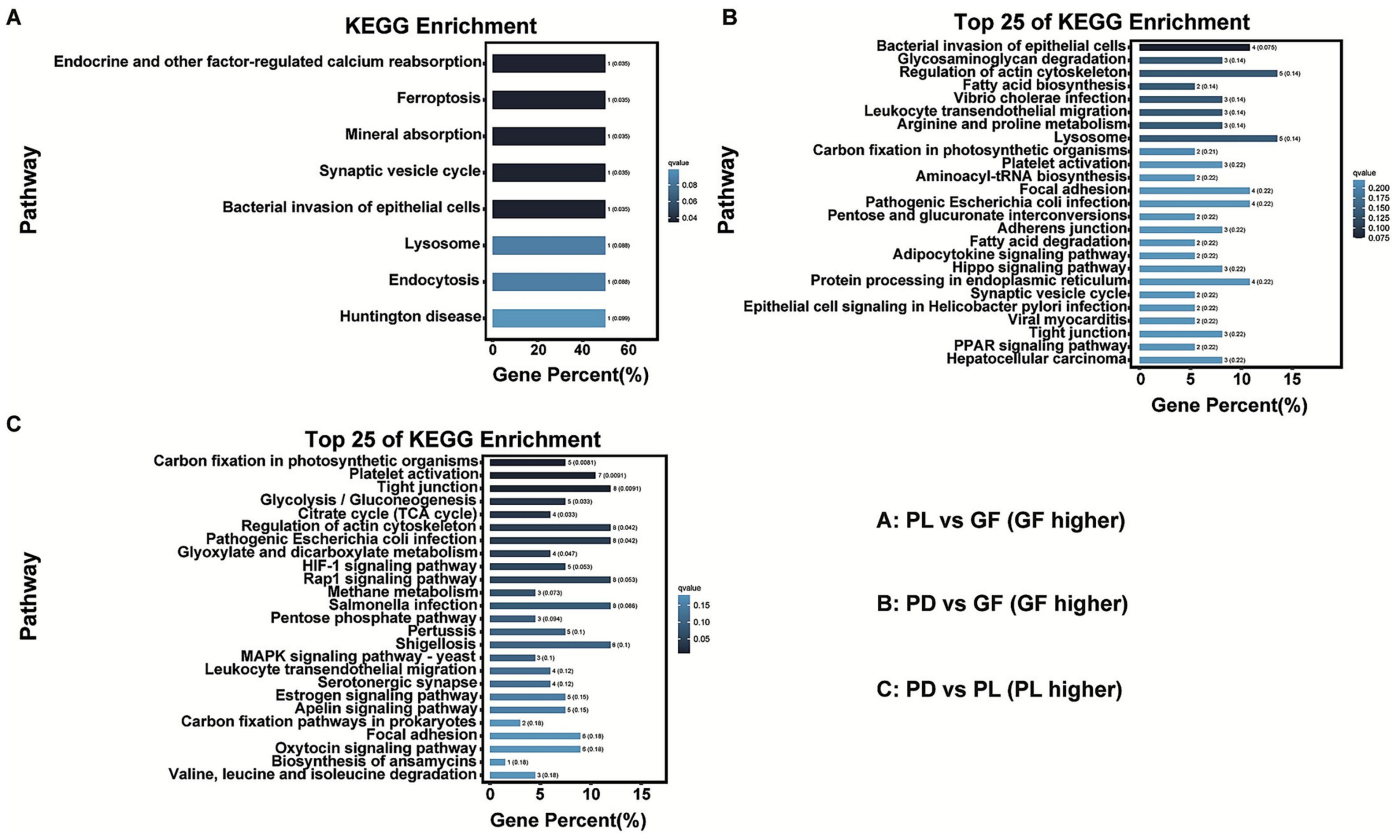
The enriched KEGG pathways among three coral host. **(A)** The top 8 KEGG pathways enriched in GF compared to PL. **(B)** The top 25 KEGG pathways enriched in GF compared to PD. **(C)** The top 25 KEGG pathways enriched in PL compared to PD.

To assess functional enrichment, KEGG pathway analyses were conducted on DEGs/DEPs with concordant changes. For the coral host, 8 pathways displayed elevated basal levels and 33 pathways had decreased basal levels in GF relative to PL. Compared with PD, GF had 110 pathways with elevated basal levels and 26 with decreased basal levels, while PL had 239 pathways with elevated basal levels and 20 with decreased basal levels. The pathway related to regulation of the actin cytoskeleton was most enriched in GF and PL compared to PD (Figure 7; Supplementary Table S13). Furthermore, the pathway involved in lysosome was also enriched in GF compared to PD, and the pathways named tight junction, pathogenic *Escherichia coli* infection, Rap1 signaling pathway, salmonella infection, and shigellose were simultaneously enriched in PL compared to PD. Particularly, the pathway involved in arginine and proline metabolism was enriched in both PL and GF compared with PD, and some pathways related to the metabolism of methionine, tryptophan, alanine, serine, valine and isoleucine were also enriched in PL compared to PD (Supplementary Figure S5; Supplementary Table S14).

## 4 Discussion

Coral susceptibility to environmental stress was related to environmental memory, growth form, and coral colony morphology. Specifically, pre-stressed corals performed higher resistance to environmental stress (Hackerott et al., 2021). Massive corals, characterized by thicker tissue layers, generally have higher tolerance to environmental stress (Qin et al., 2020). In contrast, branching corals typically show heightened susceptibility to environmental stress (Ashey et al., 2024). Here, we use multi-omics approach combined with microbial communities to explore the molecular characters of different susceptibility to environmental stress among PD, PL and GF.

### 4.1 The microbial patterns across PD, PL, and GF

Bacterial microbiome members contribute to coral health through a series of processes including sulfur cycling, nitrogen fixation, and protection against pathogens (Ziegler et al., 2019), and coral associated microbial communities were relevant to their susceptibility and resistance to environmental stressors (Tong et al., 2020). Coral reef ecosystems are experiencing an increasing decline, which highlights the importance of investigating the relationship between coral hosts and their associated bacterial communities. In the present study, we found that the alpha diversity of bacterial communities associated with PD was significantly lower than that of PL and GF. A diverse and healthy microbiome is essential to a host’s survival under changing environmental conditions (Mueller and Sachs, 2015). While previous studies have shown that PD exhibit increased microbial diversity during bleaching (Wei et al., 2024), the relatively low diversity of the bacterial community in healthy PD may result in a greater vulnerability to environmental stress. Further studies have revealed that the diversity of the coral microbiome does not correlate with disease susceptibility. Instead, a few dominant microbial taxa in the microbiome are predictors of coral disease susceptibility (Shivananda Murthy and Klein-Seetharaman, 2025). The genus *Acinetobacter* is considered both an effective first line of defense (Santos et al., 2015; Leite et al., 2017; Leite et al., 2018) and a potential pathogen (Shnit-Orland and Kushmaro, 2009; Sweet et al., 2013; Li et al., 2014; Meyer et al., 2016); additionally, high temperatures enable it to assume a core role in the coral microbiome (Sun et al., 2022). *Ralstonia* was found co-localized within the endosymbiotic dinoflagellates and gastrodermal cells of the coral host (Ainsworth et al., 2015), it was also discovered to be an opportunistic pathogen and its abundance increased significantly after *Vibrio* infection (Sun X. et al., 2023). *Sphingomonas* is an intracellular core bacterium in Symbiodiniaceae cells within coral reef (Maire et al., 2021). Additionally, some taxa within this genus are also potential coral pathogens, and unidentified species of *Sphingomonas* have been found to may be involved in “white plague,” leading to coral tissue degradation at an abnormal rate (Richardson et al., 1998; Chen et al., 2021). Notably, the abundances of *Acinetobacter*, *Ralstonia* and *Sphingomonas* were all higher in PD than in PL and GF. Previous studies have shown that several strains of *Ruegeria* had antibiotic activities against *Vibrio coralliilyticus* (Miura et al., 2019). Mohamed et al. found that *Chlorobium* was highly abundant in healthy coral tissues and could help hosts maintain their physiological functions under environmental stress (Mohamed et al., 2023). Accordingly, the abundances of both *Ruegeria* and *Chlorobium* were higher in PL and GF than in PD. These bacteria may be responsible for maintaining PL and GF’s healthy state, which accounts for their greater tolerance to environmental stress. *Endozoicomonas* was much more abundant in PD than that in the other two coral species, this bacterium is important for coral metabolism, including rapid skeleton growth, while his bacterium provides growth benefits to corals at the cost of reducing their immune defense (Shivananda Murthy and Klein-Seetharaman, 2025). Compared with PD, PL and GF exhibit consistent directional changes in top 11 genus except the *Pelagibius*. Coral associated bacteria play important roles in coral health such as nitrogen fixation, sulfur cycle, and protection against pathogens (Pollock et al., 2019), the differences in key microbial taxa between PD and the other two coral species may contribute to the greater stress resistance of PL and GF relative to PD.

### 4.2 Transcriptomic, proteomic and metabolomic profiling among PD, PL, and GF

GPCR was found to be up-regulated during coral bleaching (DeSalvo et al., 2008), and it was more abundant in PD compared with PL and GF in the present study. Transcription factor Maf was responsible for heterodimerizing and activating gene expression in response to oxidative and xenobiotic stress (Reitzel et al., 2008). Peroxidasin (PXDN), a component of the extracellular matrix (ECM), was significantly down-regulated during coral bleaching, whereas matrix metalloproteinase (MMP) was up-regulated, suggesting greater degradation of the ECM, which may aggravate coral bleaching during environmental stress (Shapiro, 1998). In *Montastraea faveolata* corals, MYO7A has been significantly upregulated when subjected to thermal stress (DeSalvo et al., 2008). RPL37 has been proposed to be a key gene that responds to thermal stress and bleaching (Yuan et al., 2019), and TRAF3 plays a direct role in regulating both innate and adaptive immunity (Haslun et al., 2021). The expressions of MMP, MYO7A, RPL37, and TRAF3 were congruently increased in PD in the present study. In addition, the gene expression profiles responding to heat stress of the two coral species from the same clade (Robust) and suborder (Faviina) were more similar when compared to the coral species from the complex clade (Avila-Magaña et al., 2021). Correspondingly, the expression profiles of thermal-related genes in massive corals (GF and PL) were more similar compared with the branching coral (PD). This further validates that genes related to environmental stress in corals susceptible to heat stress are usually expressed prior to the occurrence of bleaching traits. In contrast, massive corals such as *Porites* exhibit a delay in their molecular gene expression response to stress, which typically occurs at a later stage (Maor-Landaw and Levy, 2016).

Although the transcriptional response of coral to heat stress has been reported previously (Kirk et al., 2018), the transcriptomic and proteomic profiles are not concurrent necessarily. We conducted a comprehensive analysis of proteins involved in stress response pathways within coral species in order to uncover their actual functions. Previous studies have shown that ribosomal proteins, calmodulin, and FKBP12 are down-regulated during coral bleaching (DeSalvo et al., 2008). In healthy cells, FKBP12 and CaM could inhibit the activity of ryanodine receptors so that Ca^2+^ is only released from the endoplasmic reticulum during necessary Ca^2+^ signaling cases (DeSalvo et al., 2008). Thus, the lower basal expression of FKBP12 may lead to disrupted Ca^2+^ homeostasis in corals. However, FKBP12 exhibited higher basal expression in PD in the current study. The higher basal expression of FKBP12 in PD may be attributed to PD being a non-bleached coral.

Although mRNA abundance and proteome analysis provide valuable insights into how coral responds to environmental stress, an analysis of critical metabolic processes in corals would facilitate the understanding of the responses of corals to perturbations. Steroid molecules were found to play important roles in regulating processes such as growth, reproduction, development, and nutrient metabolism (Rougée et al., 2015; Schiffer et al., 2019). Moreover, Sun et al. found steroid hormone biosynthesis, unsaturated fatty acid (UFA) biosynthesis, and pyrimidine metabolism exhibit the greatest enrichment during coral bleaching (Sun F. et al., 2023). UFAs are associated with coral bleaching and their unsaturated degree influences corals’ resistance to thermal stress (Tchernov et al., 2004). High lipid levels were proposed that can delay coral bleaching and strengthen coral’s resistance to stress (Rodrigues et al., 2008; Anthony et al., 2009). Thus, the content and components of UFAs could be determined to monitor the health status of coral in the future. Pyrimidine metabolites are structural components of DNA and RNA, and their decrease may limit the synthesis and repair of DNA and RNA (Dumas et al., 2020). Here, both deoxycytidineand and L-Glutamine which were involved in pyrimidine metabolism decreased in PD compared with GF, and deoxycytidine was less abundant in PD than in PL. Decreased abundance of LysoPC may be associated with an increased risk of host mortality (Knuplez and Marsche, 2020). However, LysoPC was more abundant in PD compared with PL and GF. The function of LysoPC needs to be further explored in corals.

Organisms usually accumulate and synthesize amino acids in response to stress (Du et al., 2011). The presence of anaerobic alanine, which is the primary end-product of protein breakdown, indicates that coral is experiencing thermal stress (Ellis et al., 2014). The levels of serine, valine, isoleucine and proline were found increase during thermal stress, with proline acting as an excellent osmolyte or signaling molecule in stressful conditions (Hayat et al., 2012; Farag et al., 2018). Williams et al. found that corals produced more methionine at high temperatures (Williams et al., 2021). In coral hosts, thermal stress was coupled to a large increase in tryptophan metabolism (Hillyer et al., 2017). In the present study, pathways capable of metabolizing these amino acids were enriched in PL and GF compared to PD, which might support the high resistance of PL and GF to thermal stress. However, whether the activity of these pathways in PL and GF is increased still requires verification through further experiments.

## 5 Conclusion

Given the intensification of climate change and anthropogenic activity, it is important to explore the characters of corals’ environmental tolerance. By analyzing the bacteria communities of three coral species, we determined that the diversity of bacteria associated with PD was lower than those associated with PL and GF. Furthermore, the abundances of probiotic bacteria were greater in GF and PL than those of PD, while the abundances of pathogenic bacteria were greater in PD than those in PL and GF. We also found some thermal stress related genes, proteins and metabolites were more enriched in PD than in GF and PL. The correlation between DEGs and DEPs increased compared with the correlation between all genes and proteins, indicating the change in proteins was closed related to DEGs. Then, the concordantly changed genes (or proteins) that enriched in GF and PL were mainly involved in amino acid metabolism. These findings portend branching coral (PD) is less resilient to environmental stress than massive coral (PL and GF) at microbial and multi-omics levels. However, the response processes of branching coral and massive corals to environmental stress at the microbial and multi-omics levels remain unknown. Future research should conduct environmental stress experiments, validate the screened genes, proteins, and metabolites, conduct in-depth research on the plasticity of corals, and provide a basis for the protection and prediction of coral reefs.

## Data Availability

The datasets presented in this study can be found in online repositories. The names of the repository/repositories and accession number(s) can be found at: https://www.ncbi.nlm.nih.gov/genbank/, PRJNA1015294 and PRJNA1015295 https://ngdc.cncb.ac.cn/omix/select-edit/OMIX007431, OMIX007431 https://ngdc.cncb.ac.cn/omix/select-edit/OMIX007612, OMIX007612.

## Footnotes

1
https://github.com/jstjohn/SeqPrep

2
https://github.com/najoshi/sickle

3
https://github.com/tanghaibao/Goatools
