# Supplementary material for: Elaborating the molecular characteristics of corals’ different tolerance to environmental stress in Sanya Luhuitou based on multi-omics analysis
Source: Front Microbiol. 2026 Jan 6;16:1664176. doi: 10.3389/fmicb.2025.1664176 (PMC12815728; doi:10.3389/fmicb.2025.1664176)
Supplement: Supplementary file 4 [file Data_Sheet_1.DOCX]

**Supplementary Material**


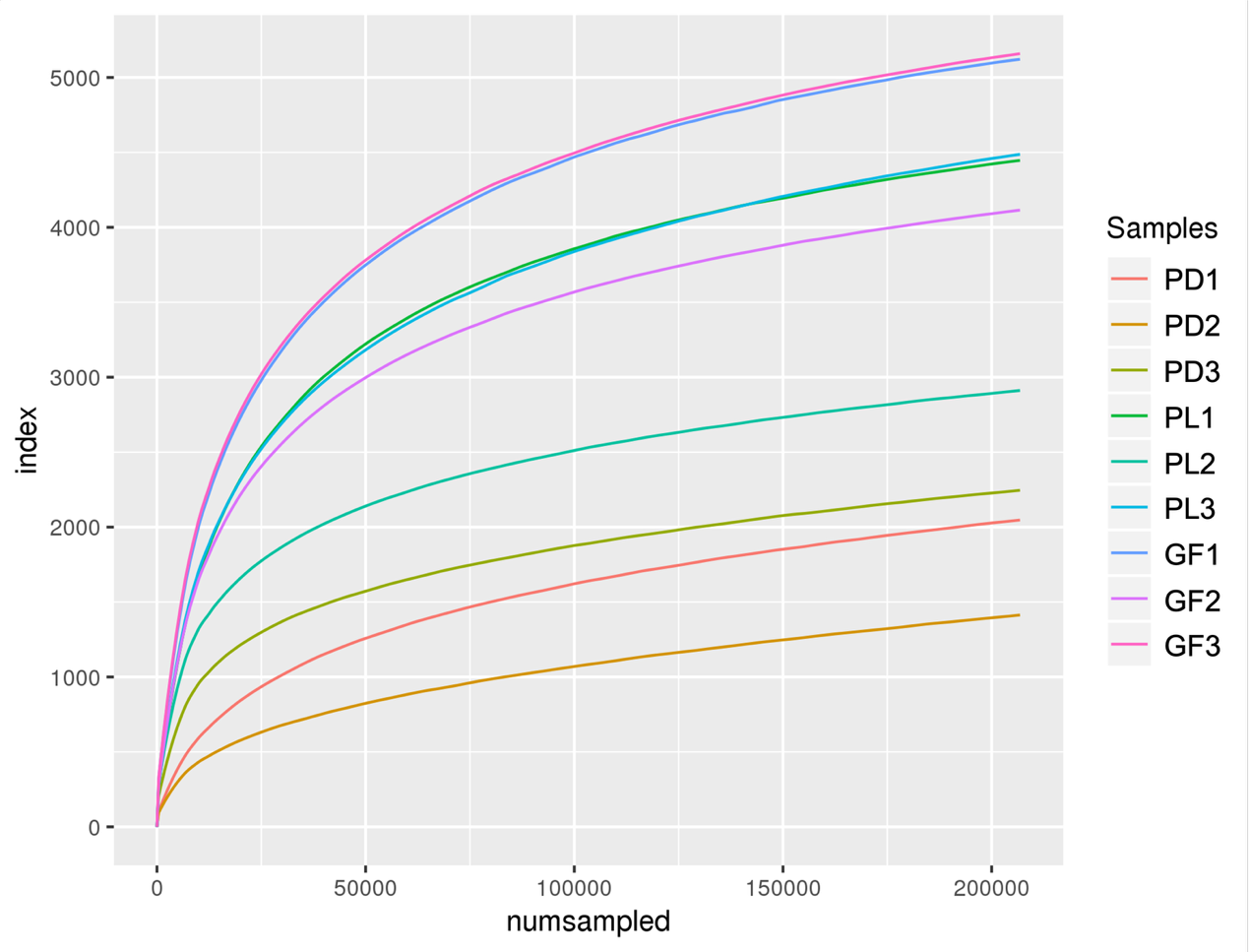


**Figure S1.** The rarefaction curve of coral samples.


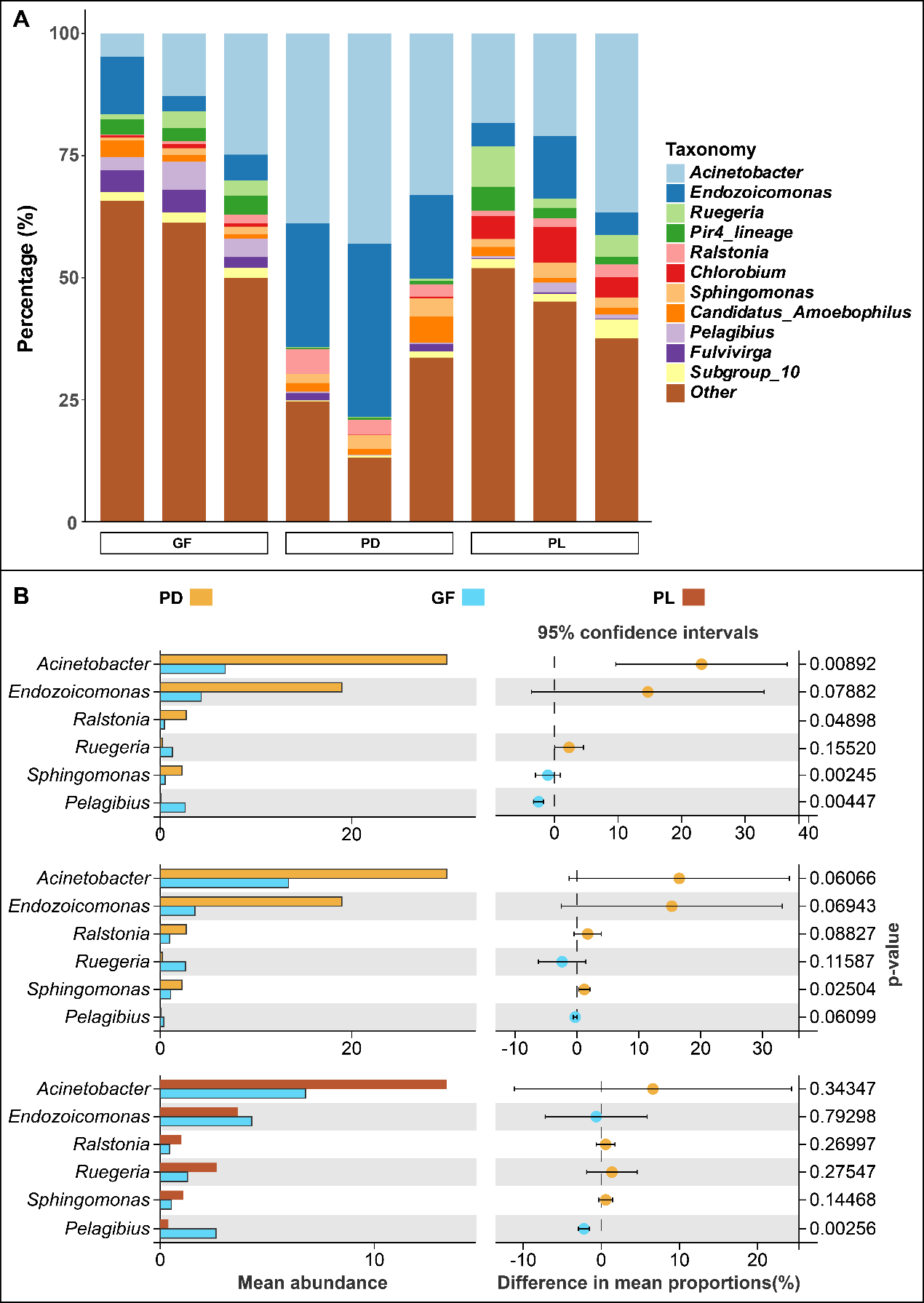


**Figure S2.** The composition of bacterial communities on genus level across PD, PL and GF. **(A)** Bacterial community composition at genus level in PD, PL, and GF. **(B)** Differences of bacterial community at genus level between PD, PL and GF.


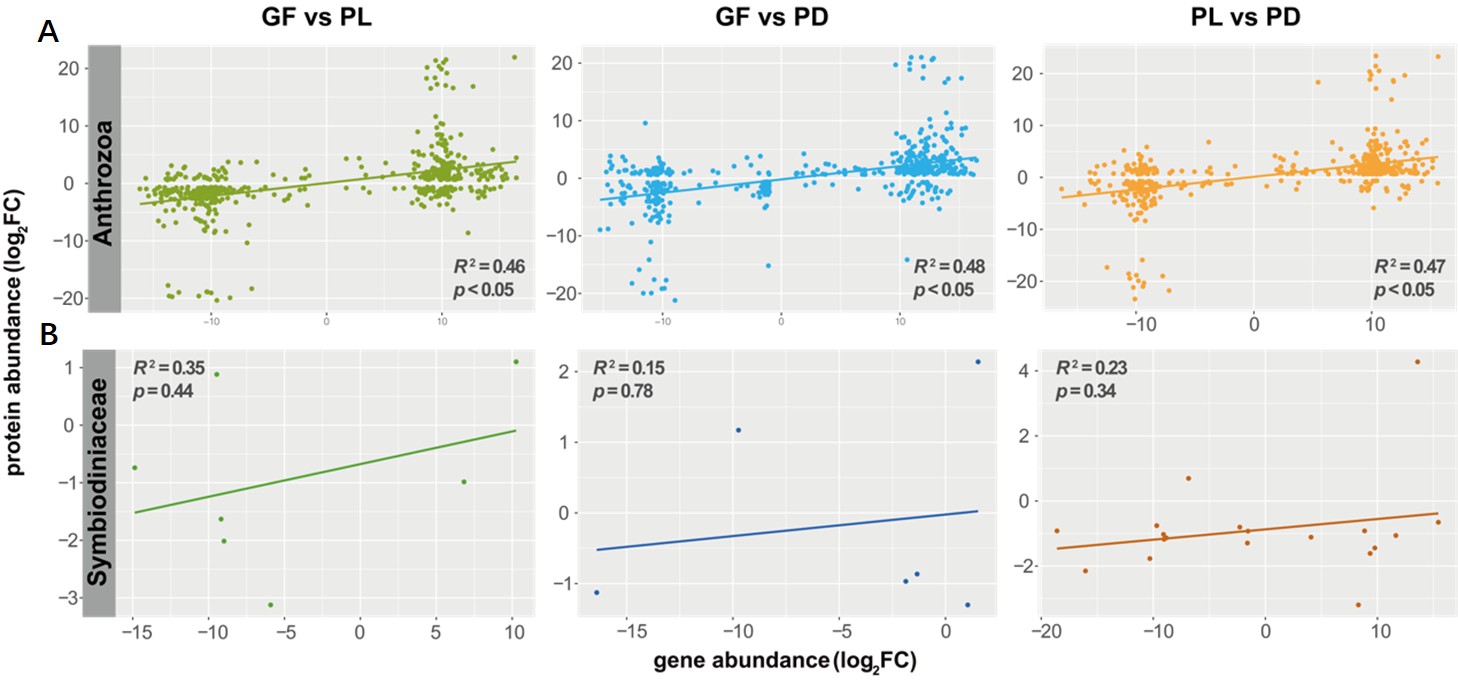


**Figure S3.** Correlation of differentially expressed genes with differentially expressed proteins for Anthozoa **(A)** and Symbiodiniaceae **(B)** of PD, PL and GF.

**
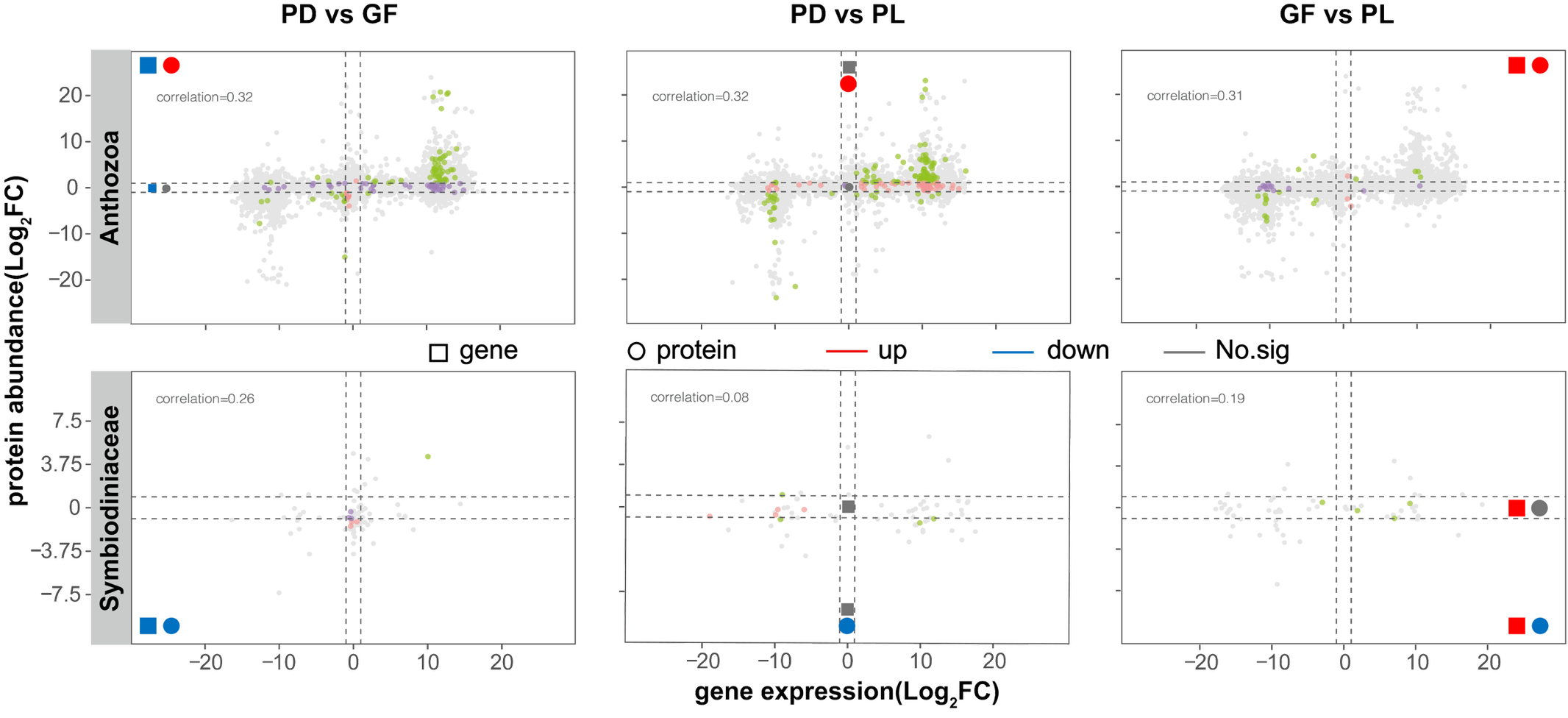
**

**Figure S4.** Scatter plot of 9-quadrant associate analyses of mRNA and proteins from log_2_FC of PD, PL and GF.


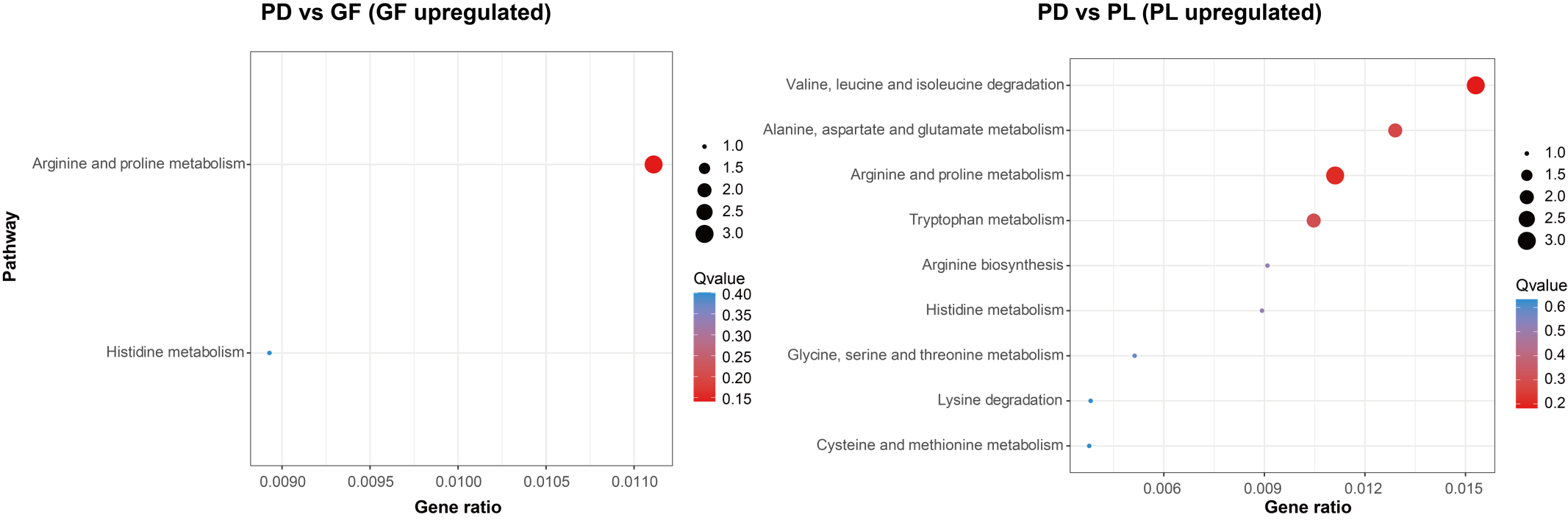
**Figure S5.** The amino acid metabolism pathways enriched in GF and PL.

**Table. S1** The reference genome of corals.

| Scientific name | Reference genome |
| --- | --- |
| Pocillopora damicornis | https://www.ncbi.nlm.nih.gov/genome/?term=Pocillopora+damicornis |
| Porites lutea | https://www.ncbi.nlm.nih.gov/datasets/genome/?taxon=51062 |
| Galaxea fascicularis | https://www.ncbi.nlm.nih.gov/datasets/genome/?taxon=46745 |

**Table. S2** Alpha diversity indexes of bacterial community associated with three coral species. Different letters indicated test results which *P* < 0.05.

|  | Simpson | Observed OTUs | Chao1 |
| --- | --- | --- | --- |
| PD | 0.8826±0.0613**^b^** | 2000.00±464.43**^b^** | 2902.89±245.50**^b^** |
| PL | 0.9732±0.0136**^a^** | 4067.33±937.39**^a^** | 4691.15±1008.21**^a^** |
| GF | 0.9856±0.0061**^a^** | 4900.67±761.68**^a^** | 5432.43±629.66**^a^** |

**Table S3** Dissimilarity tests of bacterial communities associated with three coral species (PD, PL and GF). Three different permutation tests of ANOSIM, Adonis and MRPP were performed based on Bray-Curtis distance. Bold values indicated test results which P < 0.05.

| Group | ANOSIM | | Adonis | | MRPP | |
| --- | --- | --- | --- | --- | --- | --- |
|  | R | P | R2 | P | A | P |
| PD vs GF vs PL | 1.0000 | ***0.0036*** | 0.5816 | ***0.0039*** | 0.2527 | ***0.0033*** |

**Table S4** Coral comparison and differentially expressed genes.

| **Class** | **Comparison** | **Differentially expressed genes** | **Higher basal expression genes** | **Lower basal expression genes** |
| --- | --- | --- | --- | --- |
| **Anthozoa** | PD vs PL | 82227 | 37185 | 45042 |
|  | PD vs GF | 64740 | 32838 | 31902 |
|  | PL vs GF | 83555 | 43145 | 40410 |
| **Symbiodinium** | PD vs PL | 36413 | 18453 | 17960 |
|  | PD vs GF | 1535 | 560 | 975 |
|  | PL vs GF | 36353 | 17820 | 18533 |

**Table S5** Thermal related genes with concomitant changing profiles in three coral species.

| **Gene** | **ID** | **Reference** |
| --- | --- | --- |
| Maf | Transcription factor maf | (Reitzel et al. 2008) |
| HSF1 | Heat shock transcription factor 1 | (Cleves et al. 2020) |
| NF-κB | Nuclear factor kappa B | (Schwarz et al. 2008) |
| TRAF3 | Tumor necrosis factor receptor associated factor 3 | (Haslun et al. 2021) |
| Dmbt1 | Deleted in malignant brain tumors 1 | (Avila-Magaña et al. 2021) |
| CCT7 | Chaperonin containing TCP1 subunit 7 | (Yuan et al. 2019) |
| RPS12 | Ribosomal protein S12 |  |
| DKC1 | Dyskerin pseudouridine synthase 1 |  |
| NOP56 | Nucleolar protein 56 |  |
| RPS27A | Ribosomal protein S27a |  |
| RPL37 | Ribosomal protein L37 |  |
| Tachylectin-2 | -- | (Desalvo et al. 2008) |
| TCP-1 | TCP-1 chaperonin family, zeta subunit |  |
| MYO7A | Myosin VIIA |  |
| GPCR | G protein-coupled receptor |  |
| CYLD | Cylindromatosis protein |  |
| MMP | Matrix metalloproteinase |  |
| GST-M | Glutathione s-transferase mu |  |
| CaM | Calmodulin |  |
| FKBP | FK506-binding protein |  |
| VDAC2 | Voltage-dependent anion-selective channel 2 |  |
| EF1α | Elongation factor 1α |  |
| HSP | Heat shock protein |  |
| PXDN | Peroxidasin |  |

**Table S6** Thermal related genes among three coral species.

| Gene | GeneID | log2 Ratio(GF/PD) | Profile | GeneID | log2 Ratio(PL/PD) | Profile | GeneID | log2 Ratio(GF/PL) | Profile |
| --- | --- | --- | --- | --- | --- | --- | --- | --- | --- |
| PXDN | Unigene0007170 | 10.8011 | Up | Unigene0003387 | 12.6192 | Up | Unigene0007170 | 12.7890 | Up |
|  | Unigene0020993 | 12.3132 | Up | Unigene0003968 | 9.7761 | Up | Unigene0020993 | 12.3132 | Up |
|  | Unigene0022274 | 9.3765 | Up | Unigene0025490 | -10.9085 | Down | Unigene0022274 | 10.3561 | Up |
|  | Unigene0023326 | 11.1727 | Up | Unigene0028195 | 9.9344 | Up | Unigene0023326 | 10.4793 | Up |
|  | Unigene0025490 | -10.1931 | Down | Unigene0033427 | 10.1851 | Up | Unigene0049055 | 11.2445 | Up |
|  | Unigene0049055 | 11.2445 | Up | Unigene0038223 | 9.7574 | Up | Unigene0083147 | 10.0158 | Up |
|  | Unigene0070942 | -10.5589 | Down | Unigene0054560 | 10.7361 | Up | Unigene0118148 | 9.2818 | Up |
|  | Unigene0083147 | 12.2928 | Up | Unigene0070942 | -9.7276 | Down | Unigene0118277 | -11.3547 | Down |
|  | Unigene0100710 | -12.6197 | Down | Unigene0073488 | 9.3731 | Up | Unigene0135754 | 12.7954 | Up |
|  | Unigene0118148 | 12.5144 | Up | Unigene0100710 | -8.7257 | Down | Unigene0003387 | -12.6192 | Down |
|  | Unigene0118277 | -2.4871 | Down | Unigene0118277 | 8.8676 | Up | Unigene0003968 | -13.8748 | Down |
|  | Unigene0120957 | -10.5531 | Down | Unigene0120957 | -9.8801 | Down | Unigene0028195 | -12.9344 | Down |
|  | Unigene0135754 | 12.7954 | Up | -- | -- | -- | Unigene0033427 | -10.5914 | Down |
|  | -- | -- | -- | -- | -- | -- | Unigene0038223 | -10.8618 | Down |
|  | -- | -- | -- | -- | -- | -- | Unigene0054560 | -10.4541 | Down |
|  | -- | -- | -- | -- | -- | -- | Unigene0073488 | -14.5695 | Down |
| MMP | Unigene0048899 | 9.0864 | Up | Unigene0104391 | 7.8507 | Up | Unigene0048899 | 9.2194 | Up |
|  | Unigene0039805 | -9.2618 | Down | Unigene0002688 | 11.3476 | Up | Unigene0112908 | 11.3471 | Up |
|  | Unigene0112908 | 11.3471 | Up | Unigene0039805 | -10.1712 | Down | Unigene0038875 | 10.0532 | Up |
|  | Unigene0168279 | -12.7476 | Down | Unigene0053865 | 8.9592 | Up | Unigene0097698 | 14.2274 | Up |
|  | Unigene0035085 | -8.0026 | Down | Unigene0072168 | 11.7693 | Up | Unigene0109652 | 12.3664 | Up |
|  | Unigene0079662 | -13.5134 | Down | Unigene0168279 | -12.7476 | Down | Unigene0030036 | 10.1000 | Up |
|  | Unigene0038875 | 10.9020 | Up | Unigene0035085 | -7.7965 | Down | Unigene0052890 | -10.8193 | Down |
|  | Unigene0077272 | -13.9928 | Down | Unigene0079662 | -8.2655 | Down | Unigene0114914 | 9.0611 | Up |
|  | Unigene0078265 | -11.0020 | Down | Unigene0124440 | 12.7498 | Up | Unigene0079036 | 11.3456 | Up |
|  | Unigene0097698 | 14.2274 | Up | Unigene0055031 | 9.4058 | Up | Unigene0081961 | 10.9610 | Up |
|  | Unigene0082243 | -8.6657 | Down | Unigene0077272 | -9.5378 | Down | Unigene0104391 | -12.1138 | Down |
|  | Unigene0080017 | -13.9462 | Down | Unigene0078265 | -11.0020 | Down | Unigene0002688 | -10.5230 | Down |
|  | Unigene0109652 | 9.9972 | Up | Unigene0050927 | 11.0746 | Up | Unigene0053865 | -8.8536 | Down |
|  | Unigene0030036 | 10.6931 | Up | Unigene0082243 | -9.5753 | Down | Unigene0072168 | -11.7693 | Down |
|  | Unigene0052890 | -1.7580 | Down | Unigene0033119 | 9.2909 | Up | Unigene0124440 | -12.7498 | Down |
|  | Unigene0094913 | -10.7183 | Down | Unigene0080017 | -8.9018 | Down | Unigene0055031 | -9.5872 | Down |
|  | Unigene0096286 | -8.8428 | Down | Unigene0038606 | 9.8949 | Up | Unigene0050927 | -13.5686 | Down |
|  | Unigene0085114 | -13.0624 | Down | Unigene0052890 | 9.0613 | Up | Unigene0033119 | -11.1062 | Down |
|  | Unigene0088625 | -11.3935 | Down | Unigene0094913 | -10.3668 | Down | Unigene0038606 | -10.7342 | Down |
|  | Unigene0092778 | -10.5270 | Down | Unigene0096286 | -9.3412 | Down | Unigene0052890 | -10.8193 | Down |
|  | Unigene0114914 | 13.5508 | Up | Unigene0135373 | 11.4211 | Up | Unigene0135373 | -6.4002 | Down |
|  | Unigene0029715 | -11.3086 | Down | Unigene0085114 | -9.2245 | Down | -- | -- |  |
|  | Unigene0079036 | 11.3456 | Up | Unigene0088625 | -10.3089 | Down | -- | -- |  |
|  | Unigene0081961 | 10.9610 | Up | Unigene0092778 | -11.2171 | Down | -- | -- |  |
|  | Unigene0107718 | -12.0033 | Down | Unigene0029715 | -9.2042 | Down | -- | -- |  |
|  | -- | -- | -- | Unigene0107718 | -12.0033 | Down | -- | -- |  |
| GPCR | Unigene0146841 | -1.0720 | Down | Unigene0051165 | 11.7670 | Up | Unigene0146841 | 11.9126 | Up |
|  | -- | -- | -- | Unigene0078967 | -4.3805 | Down | Unigene0051165 | -11.7670 | Down |
|  | -- | -- | -- | Unigene0146841 | -12.9846 | Down | Unigene0078967 | 3.3889 | Up |
| MYO7A | Unigene0007841 | 10.6938 | Up | Unigene0004818 | 10.6026 | Up | Unigene0007841 | 10.0639 | Up |
|  | Unigene0044548 | 13.2242 | Up | Unigene0004819 | 11.0839 | Up | Unigene0044548 | 13.2242 | Up |
|  | Unigene0097304 | -6.8071 | Down | Unigene0097304 | -11.8086 | Down | Unigene0004818 | -9.9722 | Down |
|  | Unigene0106232 | -8.5338 | Down | Unigene0106232 | -9.8096 | Down | Unigene0004819 | -13.3166 | Down |
|  | Unigene0113258 | -11.3341 | Down | Unigene0113258 | -10.4473 | Down | -- | -- |  |
| RPL37 | Unigene0015659 | 12.1033 | Up | Unigene0110292 | -8.5455 | Down | Unigene0015659 | 9.6853 | Up |
|  | Unigene0030254 | -5.6548 | Down | Unigene0148357 | -8.6294 | Down | Unigene0074798 | 9.2512 | Up |
|  | Unigene0074798 | 11.7643 | Up | Unigene0030254 | -11.6999 | Down | Unigene0110292 | 7.9670 | Up |
|  | Unigene0076818 | -9.7238 | Down | Unigene0051342 | 10.4653 | Up | Unigene0148357 | 8.7070 | Up |
|  | Unigene0144279 | -10.4234 | Down | Unigene0052946 | 10.5267 | Up | Unigene0051342 | -9.8790 | Down |
|  | -- | -- | -- | Unigene0074798 | 2.5131 | Up | Unigene0052946 | -12.3406 | Down |
|  | -- | -- | -- | Unigene0076818 | -10.4495 | Down | Unigene0158463 | 13.3592 | Up |
|  | -- | -- | -- | Unigene0144279 | -9.6716 | Down | -- | -- |  |
|  | -- | -- | -- | Unigene0158463 | -13.3225 | Down | -- | -- |  |
| TRAF3 | Unigene0005405 | -6.3122 | Down | Unigene0004151 | 9.5805 | Up | Unigene0010080 | -13.4709 | Down |
|  | Unigene0010080 | -7.3446 | Down | Unigene0004687 | 11.4887 | Up | Unigene0010546 | 8.6601 | Up |
|  | Unigene0010546 | 13.0362 | Up | Unigene0005405 | -11.3754 | Down | Unigene0014225 | 9.6954 | Up |
|  | Unigene0014225 | 12.3748 | Up | Unigene0008874 | 10.7484 | Up | Unigene0024575 | 9.3888 | Up |
|  | Unigene0024575 | 12.1418 | Up | Unigene0010080 | 6.1263 | Up | Unigene0029503 | 12.2242 | Up |
|  | Unigene0029503 | 6.7659 | Up | Unigene0011631 | 8.3910 | Up | Unigene0029504 | 11.5985 | Up |
|  | Unigene0029504 | 9.1814 | Up | Unigene0027018 | 14.5878 | Up | Unigene0048709 | 9.4341 | Up |
|  | Unigene0048709 | 8.7547 | Up | Unigene0029435 | 9.6765 | Up | Unigene0056454 | 9.8021 | Up |
|  | Unigene0056454 | 10.1866 | Up | Unigene0030120 | 11.1479 | Up | Unigene0109991 | 12.2989 | Up |
|  | Unigene0083925 | -10.8922 | Down | Unigene0083925 | -10.2158 | Down | Unigene0115340 | 12.3151 | Up |
|  | Unigene0085182 | -9.9769 | Down | Unigene0085182 | -9.1054 | Down | Unigene0004151 | -10.9033 | Down |
|  | Unigene0099703 | -9.8131 | Down | Unigene0099703 | -11.3115 | Down | Unigene0004687 | -8.1861 | Down |
|  | Unigene0109991 | 9.0662 | Up | Unigene0107918 | 8.6059 | Up | Unigene0008874 | -9.0930 | Down |
|  | Unigene0113891 | -9.3608 | Down | Unigene0113891 | -12.2868 | Down | Unigene0011631 | -9.8699 | Down |
|  | Unigene0115340 | 12.3151 | Up | Unigene0117984 | -8.9232 | Down | Unigene0027018 | -14.5878 | Down |
|  | Unigene0117984 | -13.1736 | Down | Unigene0119847 | -11.3312 | Down | Unigene0029435 | -10.1476 | Down |
|  | Unigene0119847 | -11.3312 | Down | Unigene0119848 | -11.5845 | Down | Unigene0030120 | -11.1479 | Down |
|  | Unigene0119848 | -11.5845 | Down | Unigene0120112 | 12.8587 | Up | Unigene0107918 | -12.0870 | Down |
|  | Unigene0130463 | -14.0625 | Down | Unigene0130463 | -8.7685 | Down | Unigene0120112 | -12.8587 | Down |
|  | Unigene0133982 | -10.6153 | Down | Unigene0133982 | -9.6902 | Down | -- | -- |  |
|  | Unigene0133984 | -11.8909 | Down | Unigene0133984 | -11.8909 | Down | -- | -- |  |
|  | Unigene0154845 | -6.8356 | Down | Unigene0154845 | -11.7441 | Down | -- | -- |  |
| EF1α | Unigene0101982 | 4.5209 | Up | Unigene0105330 | 2.6394 | Up | Unigene0167627 | 12.1025 | Up |
|  | Unigene0167627 | 12.1025 | Up | Unigene0052513 | 12.5884 | Up | Unigene0052513 | -7.3842 | Down |
| Maf | Unigene0049531 | -13.9176 | Down | Unigene0049531 | -8.6090 | Down | -- | -- |  |

**Table S7** Coral comparison and differentially expressed proteins.

| **Class** | **Comparison** | **Differentially expressed proteins** | **Higher basal expression proteins** | **Lower basal expression proteins** |
| --- | --- | --- | --- | --- |
| **Anthozoa** | PD vs PL | 492 | 161 | 331 |
|  | PD vs GF | 574 | 248 | 326 |
|  | PL vs GF | 449 | 242 | 207 |
| **Symbiodinium** | PD vs PL | 30 | 16 | 14 |
|  | PD vs GF | 31 | 18 | 15 |
|  | PL vs GF | 17 | 11 | 6 |

**Table S9** Thermal related proteins in three coral species.

| Protein | ProteinID | log2 Ratio(GF/PD) | Profile | ProteinID | log2 Ratio(PL/PD) | Profile | ProteinID | log2 Ratio(GF/PL) | Profile |
| --- | --- | --- | --- | --- | --- | --- | --- | --- | --- |
| Ribosomal protein S9 | Unigene0086333 | 1.1032 | Up | Unigene0086333 | 0.9462 | Up | -- | -- | -- |
|  | Unigene0086334 | 1.1032 | Up | Unigene0086334 | 0.9462 | Up | -- | -- | -- |
|  | -- | -- | -- | Unigene0151509 | -2.4070 | Down | -- | -- | -- |
| CaM | Unigene0081706 | 0.6498 | Up | Unigene0081706 | 1.0427 | Up | -- | -- | - - |
|  | Unigene0012485 | 0.5997 | Up | Unigene0012485 | 1.2911 | Up | Unigene0012485 | -0.6914 | Down |
|  | -- | -- | -- | Unigene0090882 | -0.7383 | Down | Unigene0090882 | 0.6477 | Up |
| FKBP12 | Unigene0166844 | -2.1372 | Down | Unigene0166844 | -2.7476 | Down | -- | -- | -- |

**Table S10** Coral comparison and differentially expressed metabolites.

| **Comparison** | **Differentially expressed metabolites** | **Higher basal expression metabolites** | **Lower basal expression metabolites** |
| --- | --- | --- | --- |
| PD vs PL | 778 | 321 | 457 |
| PD vs GF | 764 | 363 | 401 |
| PL vs GF | 607 | 385 | 222 |

**Table S12** Thermal related metabolites in three coral species.

| Metabolite | MetaboliteID | log2 Ratio(GF/PD) | Profile | MetaboliteID | log2 Ratio(PL/PD) | Profile | MetaboliteID | log2 Ratio(GF/PL) | Profile |
| --- | --- | --- | --- | --- | --- | --- | --- | --- | --- |
| LysoPC | NEG01645 | -2.1080 | Down | NEG01645 | -4.4271 | Down | NEG01645 | 2.3190 | Up |
|  | NEG02076 | -2.3939 | Down | NEG02076 | -4.2890 | Down | NEG02311 | 1.2642 | Up |
|  | NEG02378 | -2.0687 | Down | NEG02378 | -3.3757 | Down | -- | -- | -- |
|  | -- | -- | -- | NEG02311 | -1.4855 | Down | -- | -- | -- |
| Cholesterol sulfate | NEG1637 | -0.3315 | Down | NEG1637 | -0.3842 | DOWN | -- | -- | -- |
| Testololactone | NEG02381 | -4.4475 | Down | NEG02381 | -5.3762 | Down | POS08434 | -7.4306 | Down |
|  | -- | -- | -- | POS08434 | 22.5781 | Up | -- | -- | -- |
| Deoxycytidine | NEG05445 | 1.5980 | Up | NEG05445 | 5.4172 | Up | NEG05445 | -3.8191 | Down |
| L-Glutamine | POS08702 | 1.2692 | Up | -- | -- | -- | POS08702 | 3.1520 | Up |

**Table S14** The metabolites involved in amino acid metabolism pathways.

| Metabolites enriched in PL | Annotation | Log2Ratio(PL/PD) | Metabolites enriched in GF | Annotation | Log2Ratio(GF/PD) | Metabolites enriched in PL | Annotation | Log2Ratio(PL/GF) |
| --- | --- | --- | --- | --- | --- | --- | --- | --- |
| NEG05710 | O-Succinyl-L-homoserine | 4.9445 | POS00001 | 5-AMINOPENTANOATE | 0.8141 | NEG05710 | O-Succinyl-L-homoserine | 3.6646 |
| NEG05511 | Xanthurenic acid | 3.4848 | POS08676 | 4-Oxoproline | 1.0312 | NEG05511 | Xanthurenic acid | 3.0534 |
| NEG05124 | L-Formylkynurenine | 3.3099 | POS01825 | 4-Aminobutyraldehyde | 0.5373 | NEG05447 | 3-Hydroxyanthranilate | 4.2650 |
| NEG04117 | Anthranilate | 1.5537 | POS08480 | L-Ornithine | 1.2955 | POS08460 | L-Glutamate | 5.0390 |
| NEG05447 | 3-Hydroxyanthranilate | 8.9440 | POS02515 | Histamine | 1.1464 | POS08508 | L-Aspartate 4-semialdehyde | 6.9125 |
| POS08480 | L-Ornithine | 1.3181 | -- | -- |  | POS01024 | 3-Phosphonooxypyruvate | 0.2282 |
| POS08460 | L-Glutamate | 4.9328 | -- | -- |  | POS00011 | Creatine | 5.3302 |
| POS08508 | L-Aspartate 4-semialdehyde | 6.9428 | -- | -- |  | POS07716 | L-Leucine | 1.5416 |
| POS01024 | 3-Phosphonooxypyruvate | 0.2241 | -- | -- |  | POS02520 | 4-Aminobutyraldehyde | 0.7871 |
| POS00011 | Creatine | 6.1663 | -- | -- |  | POS08153 | Anthranilate | 4.1417 |
| POS08017 | 1-Aminocyclopropane-1-carboxylate | 1.1209 | -- | -- |  | POS00291 | Kynurenic acid | 4.0720 |
| POS07716 | L-Leucine | 2.3646 | -- | -- |  | POS00129 | Xanthurenic acid | 3.1809 |
| POS02520 | 4-Aminobutyraldehyde | 1.0177 | -- | -- |  |  |  |  |
| POS08153 | Anthranilate | 4.5329 | -- | -- |  |  |  |  |
| POS00291 | Kynurenic acid | 7.0013 | -- | -- |  |  |  |  |
| POS00129 | Xanthurenic acid | 5.7446 | -- | -- |  |  |  |  |

Reference:

Avila-Magaña, Viridiana, Bishoy Kamel, Michael DeSalvo, Kelly Gómez-Campo, Susana Enríquez, Hiroaki Kitano, Rori V. Rohlfs, Roberto Iglesias-Prieto, and Mónica Medina. 2021. 'Elucidating gene expression adaptation of phylogenetically divergent coral holobionts under heat stress', *Nature Communications*, 12: 5731.

Cleves, Phillip A., Amanda I. Tinoco, Jacob Bradford, Dimitri Perrin, Line K. Bay, and John R. Pringle. 2020. 'Reduced thermal tolerance in a coral carrying CRISPR-induced mutations in the gene for a heat-shock transcription factor', *Proceedings of the National Academy of Sciences*, 117: 28899-905.

Desalvo, M. K., C. R. Voolstra, S. Sunagawa, J. A. Schwarz, J. H. Stillman, M. A. Coffroth, A. M. Szmant, and M. Medina. 2008. 'Differential gene expression during thermal stress and bleaching in the Caribbean coral Montastraea faveolata', *Molecular Ecology*, 17: 3952-71.

Haslun, Joshua A., Briana Hauff-Salas, Kevin B. Strychar, James M. Cervino, and Nathaniel E. Ostrom. 2021. "Variation in Immune-Related Gene Expression Provides Evidence of Local Adaptation in Porites astreoides (Lamarck, 1816) between Inshore and Offshore Meta-Populations Inhabiting the Lower Florida Reef Tract, USA." In *Water*.

Reitzel, Adam M., James C. Sullivan, Nikki Traylor-knowles, and John R. Finnerty. 2008. 'Genomic Survey of Candidate Stress-Response Genes in the Estuarine Anemone Nematostella vectensis', *The Biological Bulletin*, 214: 233-54.

Schwarz, Jodi A., Peter B. Brokstein, Christian Voolstra, Astrid Y. Terry, David J. Miller, Alina M. Szmant, Mary Alice Coffroth, and Mónica Medina. 2008. 'Coral life history and symbiosis: Functional genomic resources for two reef building Caribbean corals, Acropora palmata and Montastraea faveolata', *BMC Genomics*, 9: 97.

Yuan, Jigui, Li Liu, Yanping Zhang, Cheng Shen, and Shunduo Lin. 2019. 'Molecular processes and hub genes of Acropora Palmata in response to thermal stress and bleaching', *Journal of Coastal Research*, 35: 26-32.
